# *n*-3 Polyunsaturated Fatty Acids and Metabolic Syndrome Risk: A Meta-Analysis

**DOI:** 10.3390/nu9070703

**Published:** 2017-07-06

**Authors:** Xiao-fei Guo, Xin Li, Meiqi Shi, Duo Li

**Affiliations:** 1 Department of Food Science and Nutrition, Zhejiang University, Hangzhou 310058, China; gxf@zju.edu.cn; 2 Department of Nutrition and Food Hygiene, School of Public Health, Ningxia Medicine University, Yinchuan 750004, China; 18392089377@163.com (X.L.); shimeiqi@zju.edu.cn (M.S.); 3 Institute of Nutrition and Health, Qingdao University, Qingdao 266071, China

**Keywords:** *n*-3 polyunsaturated fatty acids, docosapentaenoic acid, docosahexaenoic acid, metabolic syndrome, meta-analysis

## Abstract

The associations between *n*-3 polyunsaturated fatty acids (PUFAs) and metabolic syndrome (MetS) risk have demonstrated inconsistent results. The present study aimed to investigate whether higher circulating *n*-3 PUFAs and dietary *n*-3 PUFAs intake have a protective effect on MetS risk. A systematic literature search in the PubMed, Scopus, and Chinese National Knowledge Infrastructure (CNKI) databases was conducted up to March 2017. Odd ratios (ORs) from case-control and cross-sectional studies were combined using a random-effects model for the highest versus lowest category. The differences of *n*-3 PUFAs between healthy subjects and patients with MetS were calculated as weighted mean difference (WMD) by using a random-effects model. Seven case-control and 20 cross-sectional studies were included. A higher plasma/serum *n*-3 PUFAs was associated with a lower MetS risk (Pooled OR = 0.63, 95% CI: 0.49, 0.81). The plasma/serum *n*-3 PUFAs in controls was significantly higher than cases (WMD: 0.24; 95% CI: 0.04, 0.43), especially docosapentaenoic acid (DPA) and docosahexaenoic acid (DHA). However, no significant association was found between dietary intake of *n*-3 PUFAs or fish and MetS risk. The present study provides substantial evidence of a higher circulating *n*-3 PUFAs associated with a lower MetS risk. The circulating *n*-3 PUFAs can be regarded as biomarkers indicating MetS risk, especially DPA and DHA.

## 1. Introduction

Metabolic syndrome (MetS) refers to a combination of three or more different components of cardiometabolic risk factors, including central obesity, hypertension, dyslipidemia (elevated triglyceride (TG) and low high-density lipoprotein cholesterol concentrations), and impaired glucose tolerance [1]. Considering that patients with MetS have shown to be a predictor of type 2 diabetes mellitus (T2DM) and cardiovascular diseases (CVD) [2,3,4], it is important and necessary to curb the incidence of MetS at a preclinical stage. The mechanisms responsible for the initiation of MetS are genetics and lifestyle factors, and an effective therapeutic strategy in MetS patients could be achieved by modest weight control and regular physical activity [5]. In clinical practice, drug therapy such as statins, fibrates, and nicotinic acid has proved to achieve recommended goals if lifestyle changes are not sufficient. Considering the side-effect of pharmacological therapy, nutritional interventions have received considerable attention, especially *n*-3 polyunsaturated fatty acids (PUFAs) of marine origin.

The conclusions from epidemiological studies have demonstrated inconsistent associations between *n*-3 PUFAs and MetS risk. A prospective cohort study has suggested a null association between dietary *n*-3 PUFAs intake and MetS risk [6], whereas another prospective cohort study has indicated an inverse association [7]. Two prospective cohort studies investigated the association of circulating fatty acids with MetS risk, and the results have suggested null [8] and inverse associations [9]. Meanwhile, Several cross-sectional and case-control studies have indicated that plasma/serum *n*-3 PUFAs was significantly higher in healthy subjects compared with patients with MetS [10,11,12,13], while some studies have suggested opposite and null associations [8,13,14,15,16,17,18].

A recent meta-analysis of prospective studies has reported that dietary intake of fish and *n*-3 PUFAs was significantly inversely associated with MetS risk, respectively. However, the pooled estimate of cross-sectional studies has shown a null association [19]. Considering limitations of dietary record, the association of dietary *n*-3 PUFAs and fish intake with MetS risk might obtain imprecise and/or incorrect result. Plasma/serum fatty acids can reflect dietary fat intake and are regarded as reliable biomarkers. To clarify the relationship between circulating *n*-3 PUFAs and MetS risk, we carried out the present meta-analysis. Meanwhile, the studies that investigated the association of dietary *n*-3 PUFAs and fish intake with MetS risk were also included.

## 2. Methods

### 2.1. Study Selection

A systematic literature search was conducted in the Chinese National Knowledge Information (CNKI), PubMed, and Scopus databases up to March 2017. Fatty acid, fish, fish oil, linolenic acid, eicosapentaenoic acid (EPA), docosapentaenoic acid (DPA), docosahexaenoic acid (DHA), omega-3, and *n*-3 were paired with metabolic syndrome as search terms. Using Google and Baidu Scholar, manual searches were also scrutinized from recent meta-analyses, reviews, and original research articles.

### 2.2. Inclusive Criteria

Cross-sectional and case-control studies were included in the present meta-analysis. The outcome of interest was MetS. The original studies, which provided odd ratio (OR) with 95% confidence interval (CI) between circulating *n*-3 PUFAs and MetS risk, were included. Meanwhile, the studies that reported dietary intake of *n*-3 PUFAs or fish associated with MetS risk were also included. The articles excluded in the present study included in vitro studies, animal studies, prospective cohort studies, randomized controlled trials (RCTs), and non-original studies (meta-analyses, reviews, and commentaries).

### 2.3. Data Extraction

Data extraction was independently conducted by two investigators, and any discrepancy was resolved through discussion. The following information was extracted: first author, nation, publication year, mean age, sex, and sample size, multivariate adjusted OR with its CI. If the study reported the *n*-3 PUFAs both in healthy subjects and MetS patients, the means and standard deviations (SDs) of the *n*-3 PUFAs composition were extracted. If the SDs were not reported directly, they can be calculated from standard error of mean (SEM) or 95% confidence interval (CI) using the equations listed in the Cochrane handbook [20].

### 2.4. Statistical Analysis

Two types of meta-analysis were conducted. First, a meta-analysis was performed to estimate the association of circulating/dietary *n*-3 PUFAs with MetS risk. Multivariate adjusted OR and its corresponding 95% CI were logarithm transformed. Using a random-effects model, multivariate adjusted ORs for the highest category versus lowest category were pooled, weighted by the inverse of their variance. Second, a meta-analysis of cross-sectional and case-control studies, which reported the differences of dietary/circulating *n*-3 PUFAs between healthy subjects and patients with MetS, was included. The summary estimates were calculated as weighted mean difference (WMD) by using a random-effects model. Heterogeneity between studies was evaluated with *I*^2^ statistic, with cut-off points of 25%, 50%, and 75% representing low, moderate, and high degrees of heterogeneity. To investigate whether any one study exerted potential bias on the summary estimates, a sensitivity analysis was conducted. Publication bias was examined by visual inspection of a funnel plot and Egger’s regression test (*p* < 0.1). Statistical analysis was performed with STATA 11.0 (Stata CORP, College Station, TX, USA). A *p*-value ≤ 0.05 was considered statistically significant.

## 3. Results

### 3.1. Literature Search

**Figure 1:**
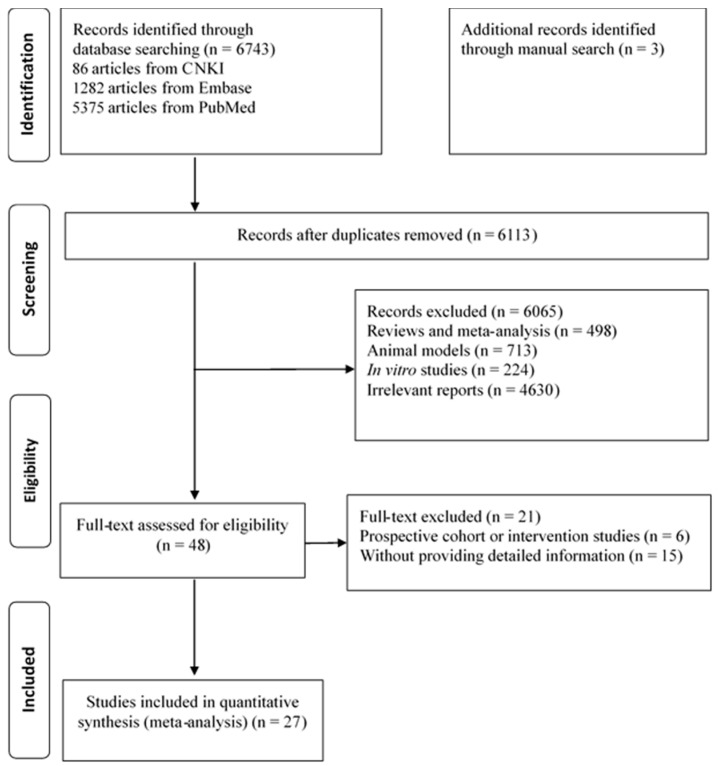
Flow chart of the study selection process.

The process of literature search is shown in Figure 1. There were 6113 articles identified from Chinese National Knowledge Information (CNKI), PubMed, and Scopus after deleting duplicates. After ruling out reviews and meta-analyses, in vitro and animal studies, and irrelevant studies, 48 citations remained. Of the 48 articles examined by full-text, an additional 21 articles were excluded for various reasons (e.g., studies without providing *n*-3 PUFAs composition, studies from prospective cohort, or intervention studies). Finally, 27 studies were eligible for the present study, including 13 studies form Europe [8,9,13,14,15,17,21,22,23,24,25,26,27], 10 studies from Asia [10,11,12,18,28,29,30,31,32,33], three studies from North America [16,34,35], and one study from South America [36] (Table S1).

### 3.2. The Association of n-3 PUFAs with MetS Risk

**Figure 2:**
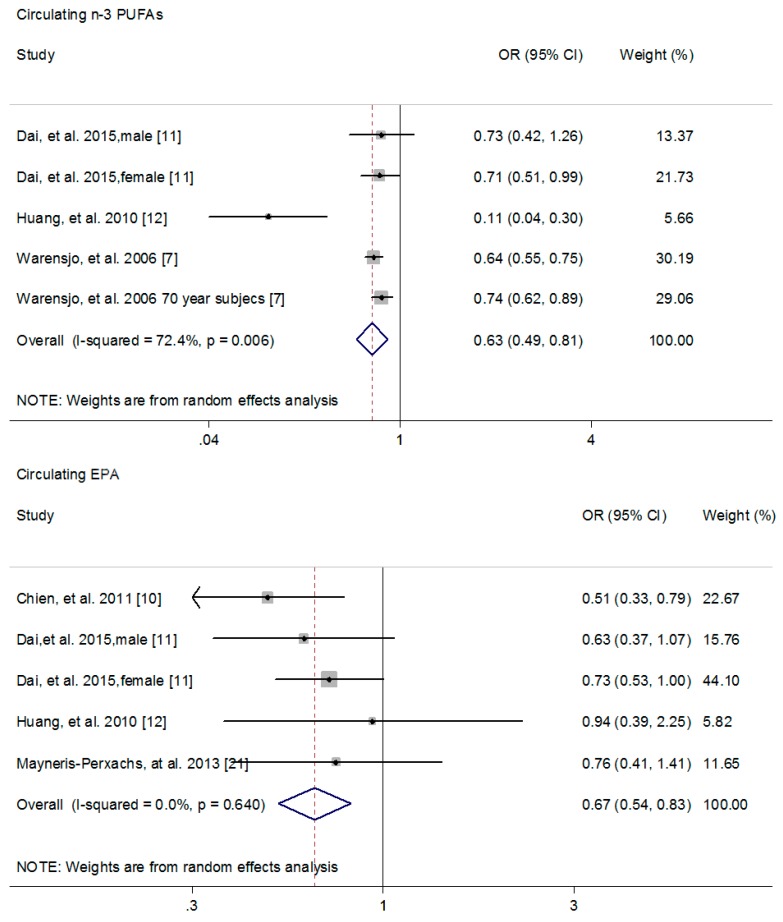
Forest plot to quantify the association of circulating *n*-3 PUFAs or EPA with metabolic syndrome risk. The pooled ORs were calculated by using a random-effects model for the highest versus lowest category. The diamonds denote summary risk estimate, and horizontal lines represent 95% CI. Abbreviations: EPA, eicosapentaenoic acid; PUFA, polyunsaturated fatty acid; OR, odd ratio.

**Figure 3:**
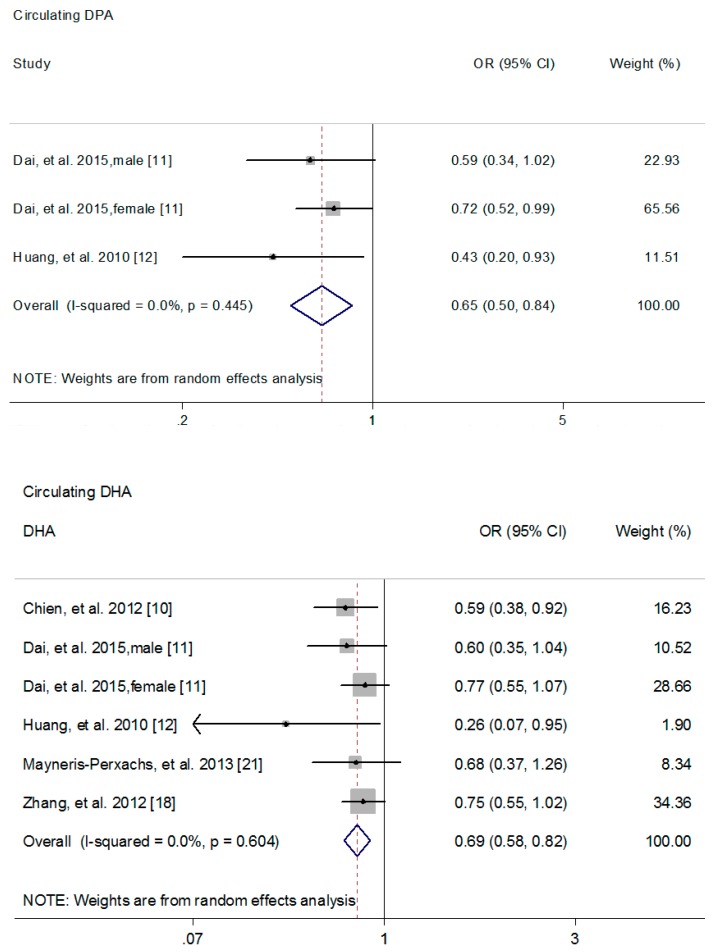
Forest plot to quantify the association of circulating DPA or DHA with metabolic syndrome risk. The pooled ORs were calculated by using a random-effects model for the highest versus lowest category. The diamonds denote summary risk estimate, and horizontal lines represent 95% CI. Abbreviations: DHA, docosahexaenoic acid; DPA, docosapentaenoic acid; OR, odd ratio.

The association of circulating *n*-3 PUFAs with MetS risk was calculated in three cross-sectional studies, and a higher circulating *n*-3 PUFAs was associated with 37% reduction in MetS risk (95% CI: 0.49, 0.81; *I*^2^ = 72.4%) (Figure 2) [9,11,12]. Meanwhile, a higher circulating EPA (Pooled OR = 0.67; 95% CI: 0.54, 0.83; *I*^2^ = 0.0%) (Figure 2) [10,11,12,21], DPA (Pooled OR = 0.65; 95% CI: 0.50. 0.84; *I*^2^ = 0.0%) (Figure 3) [11,12] and DHA (Pooled OR = 0.69; 95% CI: 0.58, 0.82; *I*^2^ = 0.0%) (Figure 3) [10,11,12,18,21] was associated with 33%, 35% and 31% reduction in MetS risk, respectively. However, no significant association was found between circulating ALA and MetS risk (Pooled OR = 1.58; 95% CI: 0.89, 2.82; *I*^2^ = 86.5%) (Figure S1) [11,12,18].

**Figure 4:**
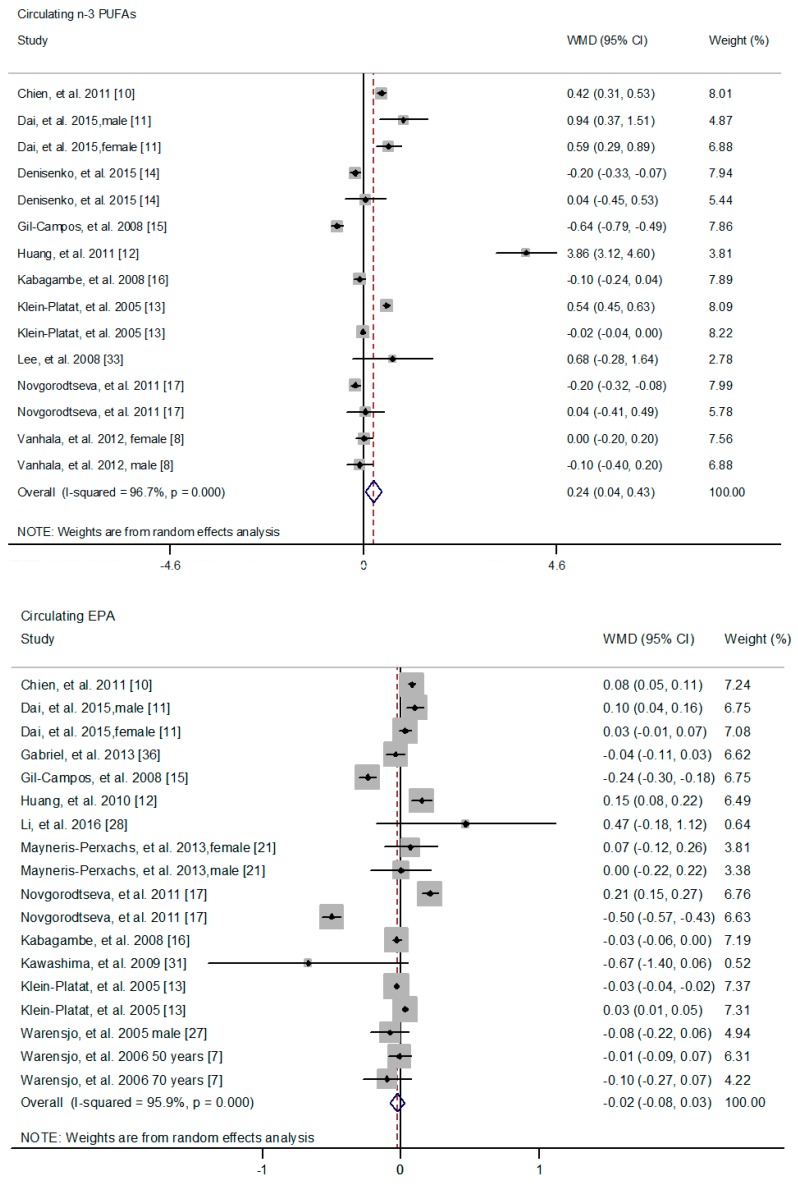
Differences of circulating *n*-3 PUFAs or EPA composition between cases and controls. The pooled effect was calculated by using a random-effects model. The diamonds denote summary risk estimate, and horizontal lines represent 95% CI. Abbreviations: EPA, eicosapentaenoic acid; PUFA, polyunsaturated fatty acid.

**Figure 5:**
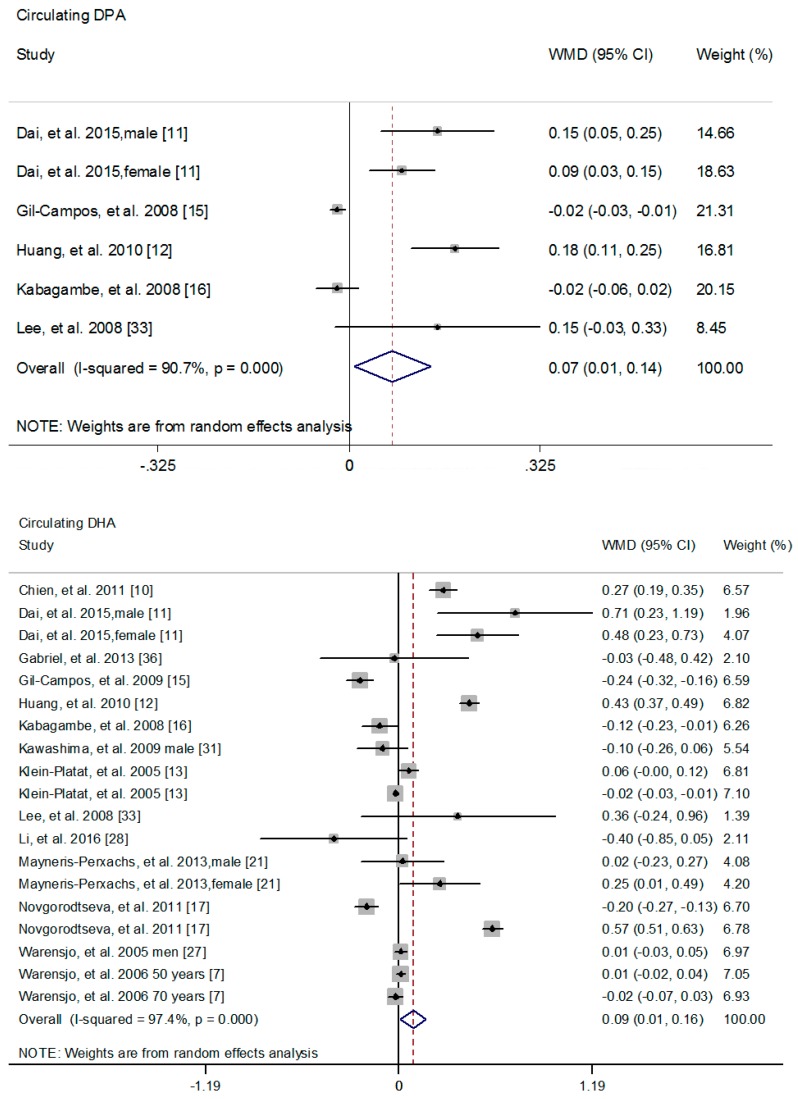
Differences of circulating DPA or DHA composition between cases and controls. The pooled effect was calculated by using a random-effects model. The diamonds denote summary risk estimate, and horizontal lines represent 95% CI. Abbreviations: DHA, docosahexaenoic acid; DPA, docosapentaenoic acid.

The difference of circulating *n*-3 PUFAs between cases and controls was also explored. No significant difference was found between cases and controls with respect to circulating ALA (WMD: 0.02; 95% CI: −0.02, 0.25) (Figure S2) [9,11,12,13,15,16,17,21,27,28,31,33,36] and EPA (WMD: −0.02; 95% CI: −0.08, 0.03) (Figure 4) [9,10,11,12,13,15,16,17,21,27,31,33,36], while circulating *n*-3 PUFAs (WMD: 0.24; 95% CI: 0.04, 0.43) (Figure 4) [8,10,11,12,13,14,15,16,17,33], DPA (WMD: 0.07; 95% CI: 0.01, 0.14) (Figure 5) [11,12,15,33] and DHA (WMD: 0.09; 95% CI: 0.01, 0.16) (Figure 5) [9,10,11,12,13,15,16,17,21,27,28,31,33,36] was significantly higher in controls compared with cases.

Four cross-sectional studies reported the association of dietary *n*-3 PUFAs intake with MetS risk [29,30,34,35]. Dietary *n*-3 PUFAs intake was associated with decreased risk of MetS, however, the summary estimate did not reach significant difference (Pooled OR = 0.84; 95% CI: 0.70, 1.01; *I*^2^ = 52.0%) (Figure S3). Seven cross-sectional studies reported dietary fish intake associated with MetS risk [22,23,24,25,26,32,34], and the summary estimate was not significant (Pooled OR = 0.94; 95% CI: 0.87, 1.02; *I*^2^ = 56.9%) (Figure S4).

Meanwhile, we investigated whether there were significant differences between cases and controls concerning dietary intake of *n*-3 PUFAs. However, no statistically significant difference was found, including dietary intake of *n*-3 PUFAs (WMD: −0.02; 95% CI: −0.13, 0.09) (Figure S5) [11,15,30,31], ALA (WMD: −0.03; 95% CI: −0.12, 0.06) (Figure S6), EPA (WMD: 1.86; 95% CI: −20.01, 23.74) (Figure S7) [11,31], DPA (WMD: 0.08; 95% CI: −8.88, 9.04) (Figure S8) [11] and DHA (WMD: 1.39; 95% CI: −3.23, 6.00) (Figure S9) [11,31].

In sensitivity analysis, we sequentially omitted one study at a time and re-calculated the remaining data. The results indicated that exclusion of any one study did not substantially influence the pooled effects (Figures S10–S14). In publication bias analysis, Egger’s regression test showed that no obvious publication bias was discerned, including *n*-3 PUFAs (*p* = 0.507), ALA (*p* = 0.317), EPA (*p* = 0.252), DPA (*p* = 0.218), and DHA (*p* = 0.260) (Figures S15–S19).

## 4. Discussion

In the present study, the associations of dietary and circulating *n*-3 PUFAs with MetS risk were systematically investigated, respectively. The original finding of the study is that a higher circulating *n*-3 PUFAs was significantly associated with decreased MetS risk when pooling the case-control and cross-sectional studies. Furthermore, the circulating *n*-3 PUFAs, especially DPA and DHA, was significantly higher in the controls, as compared with cases. However, non-significant association was found between dietary *n*-3 PUFAs or fish intake and MetS risk.

Circulating fatty acids reflect dietary fat intake from past few days to several weeks [37] and also mirrors endogenous fatty acid metabolism, for which desaturases and elongases play pivotal roles [38]. The fatty acid composition in the blood can be regarded as an indicator of dietary fat quality and as biomarkers of chronic diseases. Dietary assessment requires the participants to record or recall their food and beverage consumption over a fixed period of time. Obviously, these methods have a variety of limitations that influence their accuracy and precision, such as interviewer bias, inaccurate portion size estimations, computational errors, and incomplete food composition databases. Besides, the composition of individual fatty acids cannot be effective estimation from reported dietary intake. Due to these limitations, a growing body of researchers are considerably interested in tissue and circulating fatty acids as biomarkers indicating risk of chronic diseases [11,12,39]. The novelty of the present meta-analysis shed light on substantial evidence of a higher circulating *n*-3 PUFAs inversely associated with MetS risk. Due to imprecise and/or incorrect dietary records, non-significant association was found regarding dietary intake of *n*-3 PUFAs or fish associated with MetS risk. Thus, the present study provided substantial evidence that circulating *n*-3 PUFAs could be regarded as stable biomarkers indicating the risk of MetS.

RCTs have investigated the effect of *n*-3 PUFAs intervention on cardiometabolic risk factors in patients with MetS. A 12 week RCT was conducted in eight European countries. Supplementation with *n*-3 PUFAs (1.2 g per day) significantly reduced TG and non-esterified fatty acid concentrations [40], and the prevalence of MetS decreased by 10.5% [41]. Another RCT with fish oil (3 g per day) as intervention was conducted in women with MetS for 90 days. Supplemental fish oil significantly reduced the diastolic blood pressure compared with the baseline value. Meanwhile, the concentrations of nitric oxide (NO) and adiponectin have been increased significantly after intervention [42]. Similarly, a cross-over study has investigated whether dietary intake of white fish (Namibia hake) has beneficial effect in improving cardiovascular risk factors in MetS patients. An eight week intervention of 100 g per day of white fish showed significant lowering effects on waist circumference, diastolic blood pressure and low-density lipoprotein (LDL)-cholesterol, as compared with control group with no fish or seafood diet [43]. Another RCT found that supplementation with fish oil (3 g per day) plus olive oil (10 mL per day) significantly reduced the total cholesterol and LDL-cholesterol concentrations when compared with control group [44]. Meanwhile, few prospective cohort studies investigated the association of circulating *n*-3 PUFAs with MetS risk. A population-based cohort study was performed in Swedish men. After 20-years follow-up, circulating *n*-3 PUFAs in serum cholesteryl ester was inversely associated with MetS risk, after adjusting for smoking habits, physical activity and BMI [9], whereas another prospective cohort study from Finland found no significant association between serum *n*-3 PUFAs and MetS risk with a 6.4-year follow-up [8]. Meanwhile, two prospective cohort studies investigated the association between intake of fish and *n*-3 PUFAs and incidence of MetS [6,7]. The prospective cohort study from Korea reported that dietary intake of fish or *n*-3 PUFAs was inversely associated with risk of MetS in men, whereas no significant association was found in women [6]. Another prospective cohort study from American young adults showed that dietary intake of non-fried fish, EPA, DPA or DHA was inversely associated with MetS risk with a 25-year follow-up [7]. The RCTs and prospective studies were excluded in the present meta-analysis for two reasons. First, the published RCTs used different amount and duration of fish oil as intervention. The heterogeneous intervention might cause imprecise and/or incorrect conclusions. More importantly, due to limited date available from prospective cohort studies and few RCTs focused on the same risk factors of MstS, only cross-sectional and case-control studies were included to explore these associations.

The limitations of the study should be acknowledged. First, although a total of 27 case-control and cross-sectional studies with considerable sample-size were included, the type of these studies were regarded as a low grade of evidence. Second, fatty acid composition was measured in different biospecimens, including serum, plasma, and erythrocyte, thus the summary estimates showed significant heterogeneities when pooling the inclusive studies. Third, the present study was conducted based on observational studies, which were inevitably susceptible to inherent biases (e.g., selective biases and unknown residual confounding factors). Although the data of multivariate adjusted model were extracted, residual confounding factors might partially influence the summary estimates. Also, considering that limited studies were included, we have not performed subgroup and meta-regression analyses to explore the sources of heterogeneity. Simultaneously, several strengths of the study should be highlighted. First, *n*-3 PUFAs in the blood can accurately reflect dietary intake of fat and indicate stable biomarkers to predict future risk of MetS. Second, a great number of participants with strong statistical power provided substantial evidence of a higher circulating *n*-3 PUFAs inversely associated with MetS risk.

**Figure 6:**
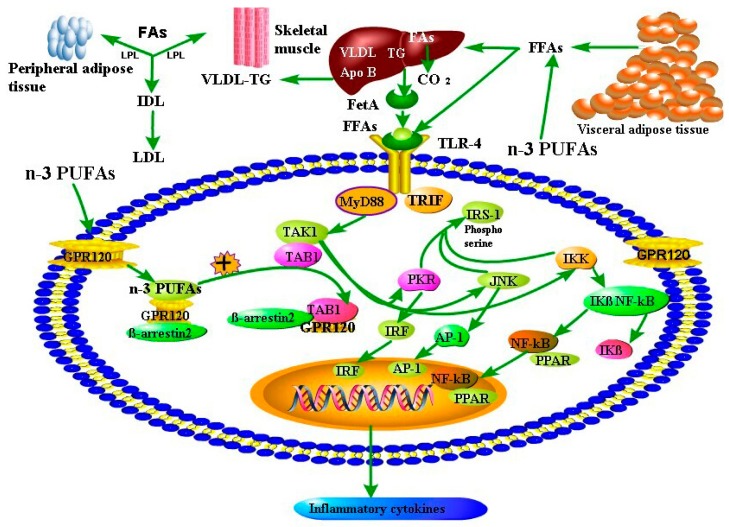
The underlying mechanisms of *n*-3 PUFAs protecting metabolic syndrome. Dysfunctions of lipid metabolism and inflammation contribute to metabolic syndrome. The high lipolytic rate in visceral adipose provides the liver with large amounts of FFAs. Impaired fat oxidation stimulates fatty acid esterification into TG, together with an augmented synthesis of Apo B, cholesterol and the secretion of VLDL. Moreover. FFAs may result in the activation of TLR4 pathways. Fet-A functions as an adaptor between FFAs and TLR4 signaling in lipid-induced inflammation. FFAs stimulate TLR-4 signaling by binding Fet-A, which then binds TLR-4. JNK, IKK, and PKR play important roles in upregulating the transcription factors (including AP-1, NF-κB, and IRF), resulting in the production of inflammatory cytokines. Moreover, these kinases can inhibit insulin signaling via serine phosphorylation of IRS-1. *n*-3 PUFAs modulate lipid and lipoprotein metabolism. Reduced VLDL production in the liver largely results from decreased availability of FFAs released from adipose stores, together with suppression of lipogenic genes and induction of genes involved in fatty acid oxidation. Inhibition of FFAs released from visceral adipose tissue due to a higher circulating *n*-3 PUFAs concentration, the TLR-4/MyD88 signaling pathway would be suppressed. Accordingly, the expression levels of PKR, IKK, JNK are inhibited. Finally, the release of inflammatory cytokines from adipocytes will be decreased. PPAR are transcription factors and regulate gene expression. PPAR are activated by non-covalent binding of ligands, such as *n*-3 PUFAs and eicosanoid mediators. Through activation of PPAR, *n*-3 PUFAs are able to regulate metabolism and other cell and tissue responses, including adipocyte differentiation and inflammation. Activation of GPR120 by *n*-3 FUFAs through binding β-arrestin 2 and TAB1 could inhibit pro-inflammatory pathways. Abbreviations: PUFA, polyunsaturated fatty acid; FFAs, free fatty acids; Apo B, apolipoprotein B; IDL, intermediate-density lipoprotein; LPL, lipoprotein lipase; TAG, triacylglycerol; FA, fatty acid; Fet-A, Fetuin-A; TLR-4, toll-like receptor 4; AP-1, activator protein-1; IKK, inhibitor of NF-κB kinase; IRF, interferon regulatory factor; NF-κB, nuclear factor-κB; IRS-1, insulin receptor substrate 1; JNK, c-jun N-terminal kinase; PKR, protein kinase R; TRIF, TIR domain-containing adapter-inducing interferon-β; MyD88, myeloid differentiation factor 88. TAK1, transforming growth factor-activated kinase 1; TAB1, transforming growth factor-β activated kinase 1 binding protein 1; PPAR, peroxisome proliferator-activated receptor; GPR120, G-protein-coupled receptor 120.

The possible mechanisms through which a higher circulating *n*-3 PUFAs predicts a lower risk of MetS mainly consist of three aspects as follows (Figure 6):

(1) The *n*-3 PUFAs have beneficial effects on fatty acid metabolism. With the release of free fatty acids from visceral adipose tissue, it will be synthesized as a form of TG. However, a higher circulating *n*-3 PUFAs reduces the release of free fatty acids from visceral adipose tissues, thus the synthesis and release of TG from liver would be inhibited [4]. Nuclear receptors (peroxisome proliferator activated receptors (PPAR) family) and transcription factors (sterol regulatory element-binding protein-1 (SREBP-1)) participate in carbohydrate and lipid synthesis and oxidation [45]. As the ligands of *n*-3 PUFAs, activation of PPAR-α/γ contributes to fatty acid oxidation in the mitochondria and reduces the synthesis of TG in the liver [46]. Meanwhile, the enzymes participating in lipogenesis, including acetyl-CoA carboxylase (ACC), fatty acid synthase (Fans), and TG synthesis (e.g., glycerol-phosphate acyltransferase), are regulated by SREBP-1c [46]. It has been reported that administration of DHA could inhibit expression level of SREBP-1c and that might be attributable to the activation of AMP-activated protein kinase (AMPK) [47,48].

(2) A higher circulating *n*-3 PUFAs improves the fluidity of cell membrane in favour of signaling transduction. Adequate fluidity of cell membrane has beneficial effect to improve the functioning of surface receptors and signal transduction [4,49]. With the improvement of membrane fluidity, it will increase insulin sensitivity and promote transduction of glucose transporter-4 (Glut-4) for glucose transport into the cytoplasm to stimulate glucose uptake, contributing to glucose homeostasis [50,51].

(3) Chronic low-grade inflammation contributes to the initiation, propagation and development of metabolic disorders, including obesity, insulin resistance, T2DM and CVD [52,53]. Accumulating evidence has suggested that *n*-3 PUFAs and their metabolites could modulate inflammatory signaling pathways, and have shown decreased production of tumor necrosis factor (TNF)-α, interleukin (IL)-1β and IL-6 in vitro and in vivo models [51,54,55,56]. The anti-inflammatory effects of *n*-3 PUFAs appears to decrease inhibitor of κB (IκB) phosphorylation, and to inhibit the activation of nuclear transcription factor-κB (NF-κB) [57,58]. G-protein coupled surface receptors (GRPs) are of importance signaling molecules for many aspects of cellular functions. Ligands bind specifically to GPRs stimulating and inducing a variety of cellular reactions via several second messenger pathways [51]. The inhibitory effect of *n*-3 PUFAs on NF-κB activation might be attributed to activation of GRP120 via *n*-3 PUFAs [59].

## 5. Conclusions

The present meta-analysis provides substantial evidence that a higher circulating *n*-3 PUFAs is associated with a significant reduction in MetS risk, whereas no significant association was found between dietary *n*-3 PUFAs or fish intake and MetS risk. Circulating *n*-3 PUFAs might be regarded as independent biomarkers in predicting the development of MetS. The evidence of the present study will have important public implications in preventing MetS through supplemental long-chain *n*-3 PUFAs of marine-origin. Furthermore, added RCTs and epidemiological studies with large sample-size are warranted to confirm the findings of this study.

## Supplementary Materials

The following are available online at www.mdpi.com/2072-6643/9/7/703/s1, Figure S1: Forest plot to quantify the association of circulating ALA composition with metabolic syndrome risk, Figure S2: Differences of circulating ALA composition between cases and controls, Figure S3: Forest plot to quantify the association of circulating dietary *n*-3 PUFA intake with metabolic syndrome risk, Figure S4: Forest plot to quantify the association of circulating dietary fish intake with metabolic syndrome risk, Figure S5: Differences of dietary *n*-3 PUFA intake between cases and controls, Figure S6: Differences of dietary ALA intake between cases and controls, Figure S7: Differences of dietary EPA intake between cases and controls, Figure S8: Differences of dietary DPA intake between cases and controls, Figure S9: Differences of dietary DHA intake between cases and controls, Figure S10: Sensitivity analysis for *n*-3 PUFAs associated with MetS risk, Figure S11: Sensitivity analysis for ALA associated with MetS risk, Figure S12: Sensitivity analysis for EPA associated with MetS risk, Figure S13: Sensitivity analysis for DPA associated with MetS risk, Figure S14: Sensitivity analysis for DHA associated with MetS risk, Figure S15: Funnel plot for circulating *n*-3 PUFAs associated with MetS risk, Figure S16: Funnel plot for circulating ALA associated with MetS risk, Figure S17: Funnel plot for circulating EPA associated with MetS risk, Figure S18: Funnel plot for circulating DPA associated with MetS risk, Figure S19: Funnel plot for circulating DHA associated with MetS risk, Table S1: Characteristics of included studies.
