# Supplementary material for: n-3 Polyunsaturated Fatty Acids and Metabolic Syndrome Risk: A Meta-Analysis
Source: Nutrients. 2017 Jul 6;9(7):703. doi: 10.3390/nu9070703 (PMC5537818; doi:10.3390/nu9070703)
Supplement: Supplementary file 1 [file nutrients-09-00703-s001.zip › nutrients-193218-supplementary.pdf]

Table S1 Characteristics of included studies

Study, year	Nation	Type of the Studies	Participants /cases	Age (years)	Gender (F/M)	Exposure assessment	Exposure categories	MetS Ascertainment	Adjusted variables
Chien, 2011	China	Case-control	1986/1000	54.7 ± 10.7	710/1276	Plasma	n-3 PUFA; EPA and DHA	NCEP ATP III	Age, gender, BMI, smoking, drinking, exercise, LDL cholesterol, systolic, diastolic blood pressure, uric acid, fasting glucose levels and total fat amount in plasma
Dai, 2015	China	Cross-sectional	720/180	57.6±6.1	Male	Erythrocyte	Total n-3 PUFA; ALA; EPA; DPA and DHA	NCEP ATP III	Age, dietary intake of total energy, saturated fat, fiber, alcohol drinking, smoking status, education level, physical activity and BMI
Dai, 2015	China	Cross-sectional	1614/558	55.4 ±5.3	Female	Erythrocyte	Total n-3 PUFA; ALA; EPA; DPA and DHA	NCEP ATP III	Age, dietary intake of total energy, saturated fat, fiber, alcohol drinking, smoking status, education level, physical activity and BMI
Denisenko, 2015	Russia	Cross-sectional	46/31	21-69	NR	Plasma erythrocytes	n-3 PUFA	AAC	
Gabriel, 2014	Mexico	Case-control	75/44	13.6 ± 1.3	37/38	Erythrocyte	ALA; EPA and DHA	a	
Gil-Campos, 2008	Spain	Case-control	37/17	9.4±0.5	11/26	plasma	n-3 PUFA; ALA; EPA DPA and DHA	NCEP ATP III	
Huang,	China	Cross-	919/210	44.4 ± 8.1	201/717	Plasma PL	n-3 PUFA;	NCEP	Age and sex

2010		sectional					EPA; DPA and DHA	ATP III	
Lai, 2013	USA	Cross-sectional	4941/1038	52.1 ± 13.9	2672/ 2269	FFQ	Dietary n-3 PUFA intake	NCEP ATP III	Age, gender, race, alcohol intake, smoking, exercise, TV watching, energy intake, multivitamin use, and fruits and vegetables intake
Lee, 2008	Korea	Case-control	132/44	54.5 ± 9.2	60/68	Erythrocyte	n-3 PUFA, ALA, EPA, DPA and DHA	NCEP ATP III	
Li, 2006	China	Case-control	195/76	60.5 ± 10.7	67/52	Serum	ALA; EPA and DHA	CDS	
Mayneris-Perxachs, 2013	Spain	Cross-sectional	427/315	67.6 ± 5.9	250/177	Plasma	ALA; EPA and DHA	AHA; WHF, IAS; IASO	Gender, age, energy intake, BMI, smoking status (current; past, 0-1 year; past, 1-5 year; past, >5 years; never), occupation (worker, unemployed or unfit, retired), and educational level (none, primary school, secondary school, university)
Mennen, 2000	France	Cross-sectional	2439/600	46.8 ± 74.0	Male	FFQ	Fish intake	Arbitrary criteria	Age, waist-hip ratio and energy intake
Mennen, 2000	France	Cross-sectional	2537/941	47.5 ± 6.1	Female	FFQ	Fish intake	Arbitrary criteria	Age, waist-hip ratio and energy intake
Mirmiran, 2012	Iran	Cross-sectional	2457/NR	F: 38.0 ± 12.8<br>M: 40.7 ± 14.4	1130/ 1327	FFQ	Dietary n-3 PUFA intake	NCEP ATP III	Age, gender, smoking status, physical activity, total energy intake, percentage of energy from carbohydrate, protein, saturated fatty acid, monounsaturated fatty acid, oleic acid, and total fiber
Noel,	USA	Cross-	1207/NR	45-75	NR	FFQ	Dietary n-3	AHA	Age, gender, smoking and alcohol use, physical

2010		sectional					PUFA intake		activity, education, fish oil supplement use, acculturation, total energy, total fat, dietary fiber, lipid-lowering medication use and BMI
Novgorodtseva,2011	Russia	Case-control	46/31	21-69	NR	Plasma	Total n-3 PUFA; ALA; EPA and DHA	AHA	
Kouki, 2011	Finland	Cross-sectional	663/182	66.3 ± 5.4	Male	4-day food record	Fish intake	NCEP ATP III	Age, smoking, alcohol consumption, education and maximal oxygen uptake
Kouki, 2011	Finland	Cross-sectional	671/169	66.4 ± 5.3	Female	4-day food record	Fish intake	NCEP ATP III	Age, smoking, alcohol consumption, education and maximal oxygen uptake
Kabagambe, 2008	USA	Cross-sectional	630/396	48.7 ± 16.2	539/491	Erythrocyte	Total n-3 PUFA; ALA; EPA; DPA and DHA	NCEP ATP III	
Kawashima, 2009	Japan	Case-control	51/24	49.8 ± 6.3	Male	Plasma	ALA; EPA and DHA	JCDCMS	
Klein-Platat, 2005	France	Cross-sectional	75/15	NR	NR	Plasma PL and CE	n-3 PUFA; ALA; EPA and DHA	NCEP ATP III	
Park, 2016	Korea	Cross-sectional	38766/8536	NR	23059/15707	24 h dietary recall	Dietary n-3 PUFA intake	NCEP ATP III	Sex, age, BMI, residence area, education level, smoking status, alcohol consumption, physical activities and obesity
Ruidavets, 2007	France	Cross-sectional	912/214	55.1 ± 6.1	Male	3-day food record	Fish intake	NCEP ATP III	Age, center, physical activity, level of education, smoking habits, alcohol intake, drugs for

									hypertension and dyslipidaemia, energy intake (without alcohol), dieting and diet quality index
Tørris, 2016	Norway	Cross-sectional	12981/2934	30-87	6880/ 6101	FFQ	Fish intake	JIS	Age, physical activity, cod liver oil, parity and lactation
Tørris, 2016	Norway	Cross-sectional	23907	44 ± 11.69	12432/ 11475	FFQ	Fish intake	JIS	Gender, education, physical activity, living with a spouse, parity and lactation
Vanhala, 2012	Finland	Cross-sectional	274/92	46.1 ± 6.0	Male	Serum	n-3 PUFA	AHA; WHF; IAS; and IASO	
Vanhala, 2012	Finland	Cross-sectional	391/111	45.8±6.2	Female	Serum	n-3 PUFA	AHA; WHF; IAS; and IASO	
Warensjö, 2006	Sweden	Cross-sectional	2009/281	50	Male	Serum CE	ALA; EPA and DHA	NCEP ATP III	BMI, smoking habits, and physical activity
Warensjö, 2006	Sweden	Cross-sectional	576/147	70	Male	Serum CE	ALA; EPA and DHA	NCEP ATP III	BMI, smoking habits, physical activity, smoking habits and physical activity
Warensjö, 2005	Sweden	Cross-sectional	706/119	70	Male	Serum CE	ALA; EPA and DHA	NCEP ATP III	
Zaribaf, 2014	Iran	Cross-sectional	420/105	35.2 ± 7.2	Female	FFQ	Fish intake	NCEP ATP III	Age, energy intake, physical activity, socioeconomic status, medication use, marital and menopausal status, dietary intakes of red meat, whole and refined grains, fruits, vegetables, legume and nuts, dairy products, fiber, oils and BMI
Zhang, 2012	China	Cross-sectional	2754/1123	50-70	NR	Erythrocyte	ALA and DHA	NCEP ATP III	Age, sex, region, residence, smoking, alcohol drinking, physical activity, educational attainment,

			family history of diabetes and CVD, total energy intake, red meat intake, fiber intake, BMI and CRP

Abbreviations: a, waist circumference (WC) > 90th percentile and at least three of the following, HDL-C < 50 mg/dL, TG > 110 mg/dL, diastolic and/or systolic blood pressure (BP) > 90th percentile adjusted by age, gender and height, and/or fasting glucose (FG) > 100 mg/dL; AAC, American Association of Cardiology; AHA, American Heart Association; BMI, body mass index; CE, Cholesteryl esters; CDS, Chinese Diabetes Society; FFQ, Food-frequency questionnaire; IAS, International Atherosclerosis Society; IASO, International Association for the Study of Obesity; JCDCMS, Japanese Committee for the Diagnostic Criteria of Metabolic Syndrome; MetS, Metabolic syndrome; NCEP-ATP III, National Cholesterol Education Program Adult Treatment Panel III criteria; NHLBI, National Heart, Lung, and Blood Institute; PL, Phospholipid; JIS, Joint Interim Societies; WHF, World Heart Federation; WHO, World Health Organization.

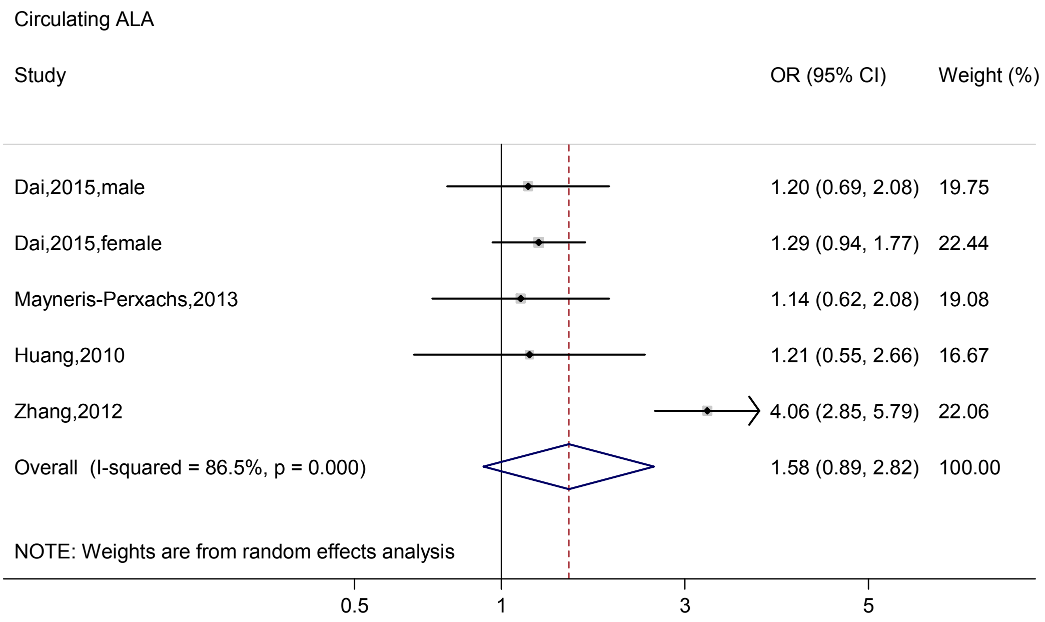

Figure S1 Forest plot to quantify the association of circulating ALA composition with metabolic syndrome risk

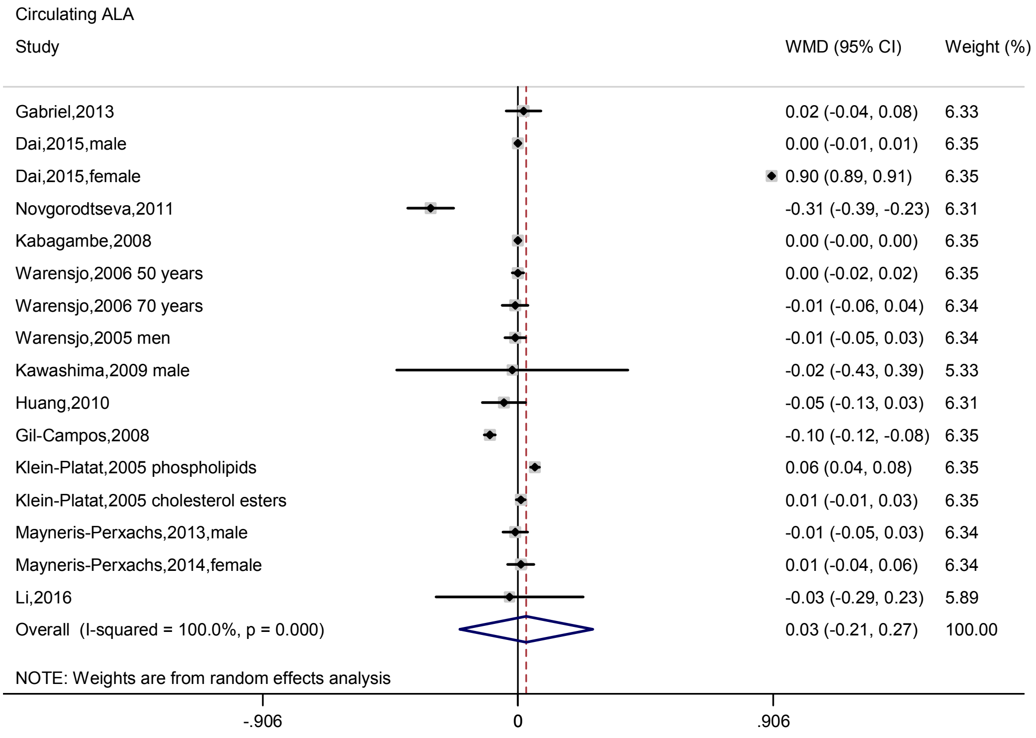

Figure S2 Differences of circulating ALA composition between cases and controls

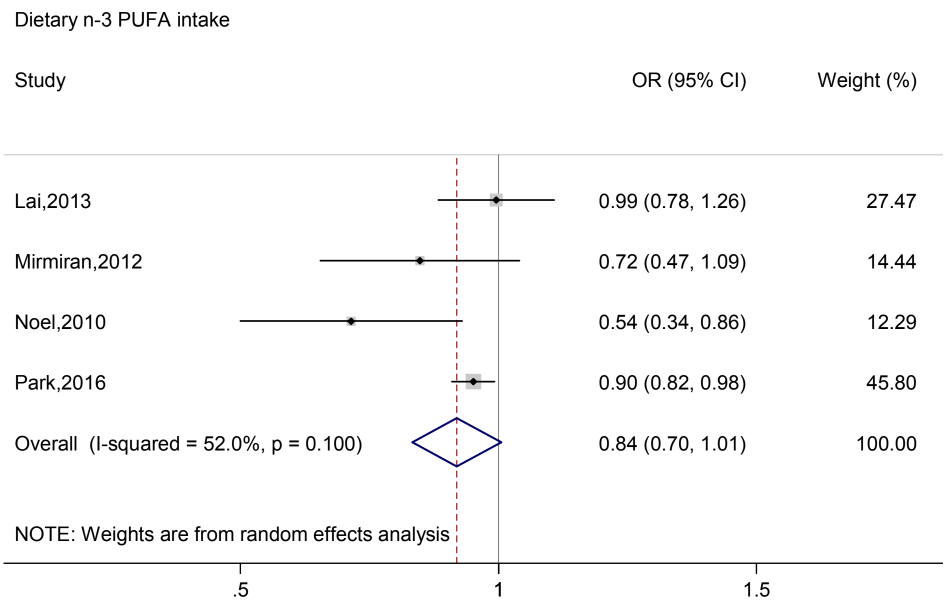

Figure S3 Forest plot to quantify the association of circulating dietary n-3 PUFA intake with metabolic syndrome risk

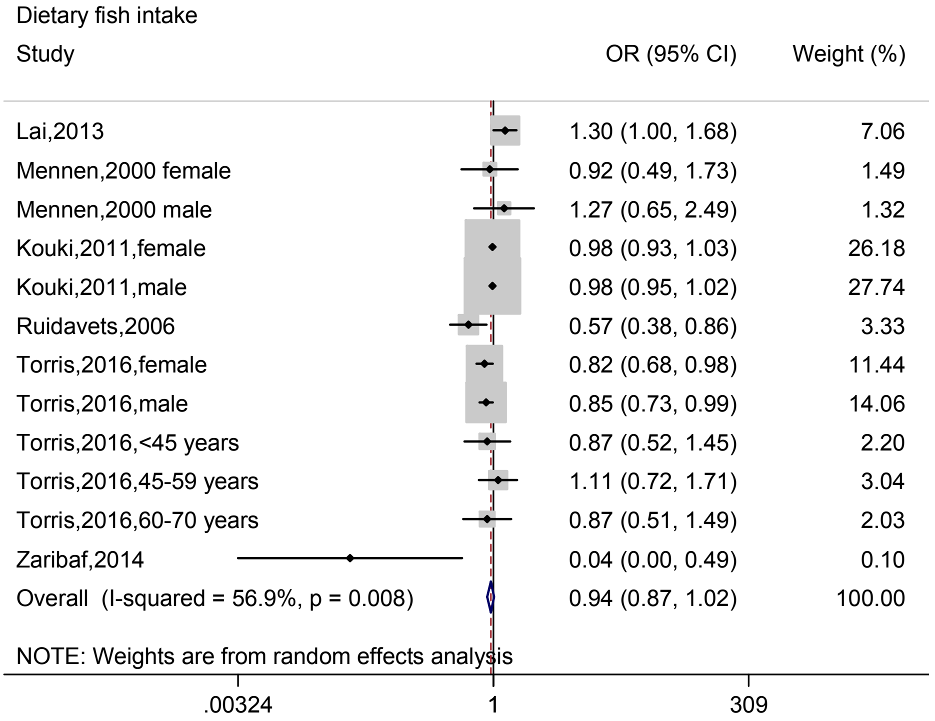

Figure S4 Forest plot to quantify the association of circulating dietary fish intake with metabolic syndrome risk

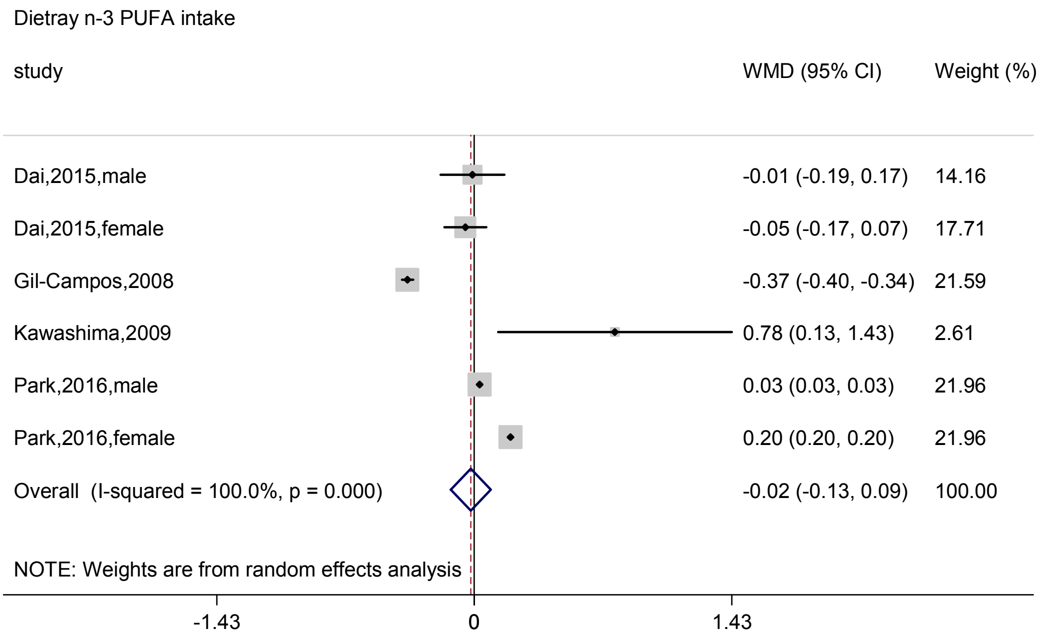

Figure S5 Differences of dietary n-3 PUFA intake between cases and controls

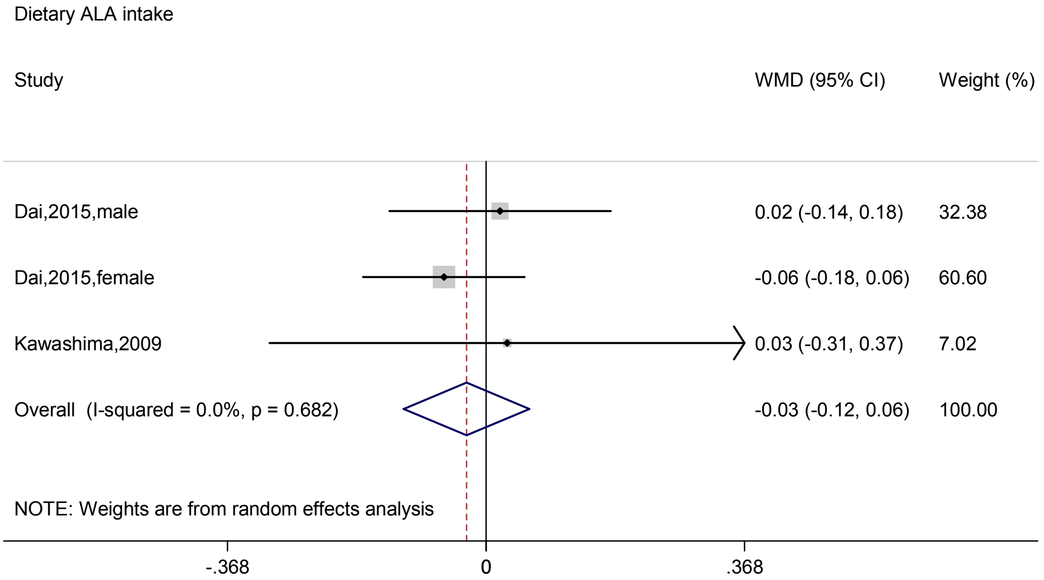

Figure S6 Differences of dietary ALA intake between cases and controls

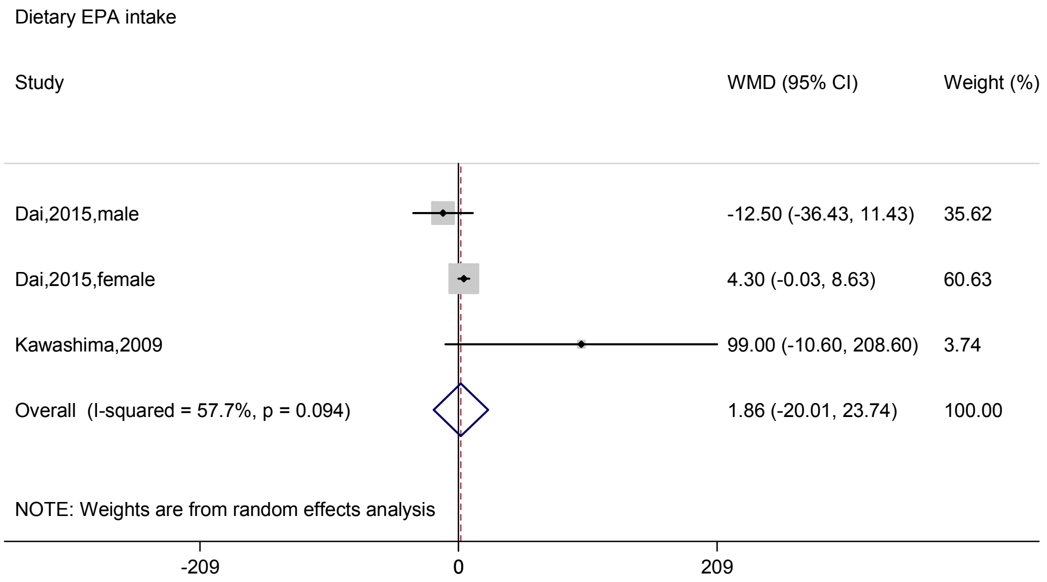

Figure S7 Differences of dietary EPA intake between cases and controls

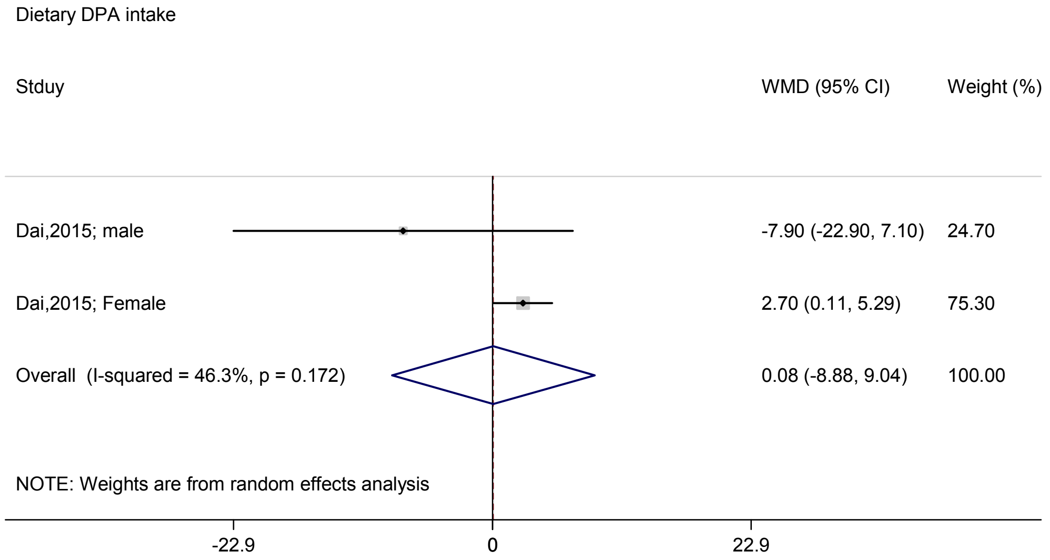

Figure S8 Differences of dietary DPA intake between cases and controls

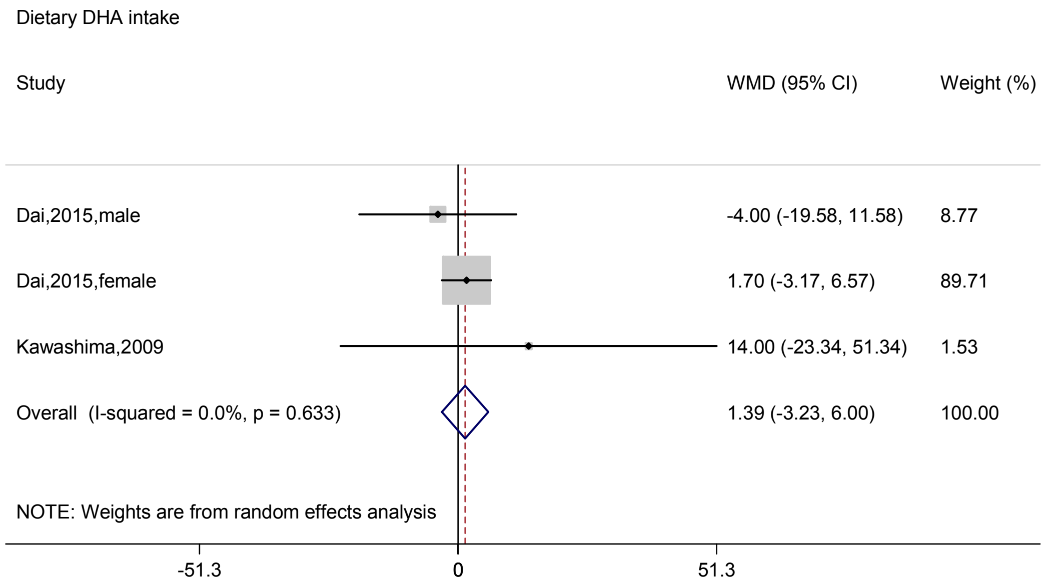

Figure S9 Differences of dietary DHA intake between cases and controls

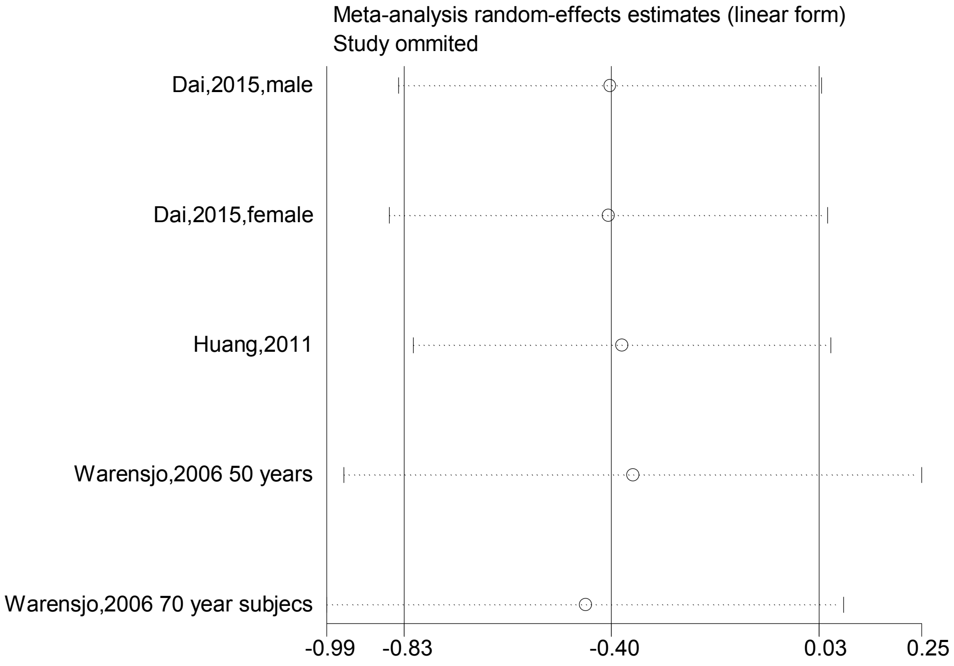

Figure S10 Sensitivity analysis for n-3 PUFAs associated with MetS risk

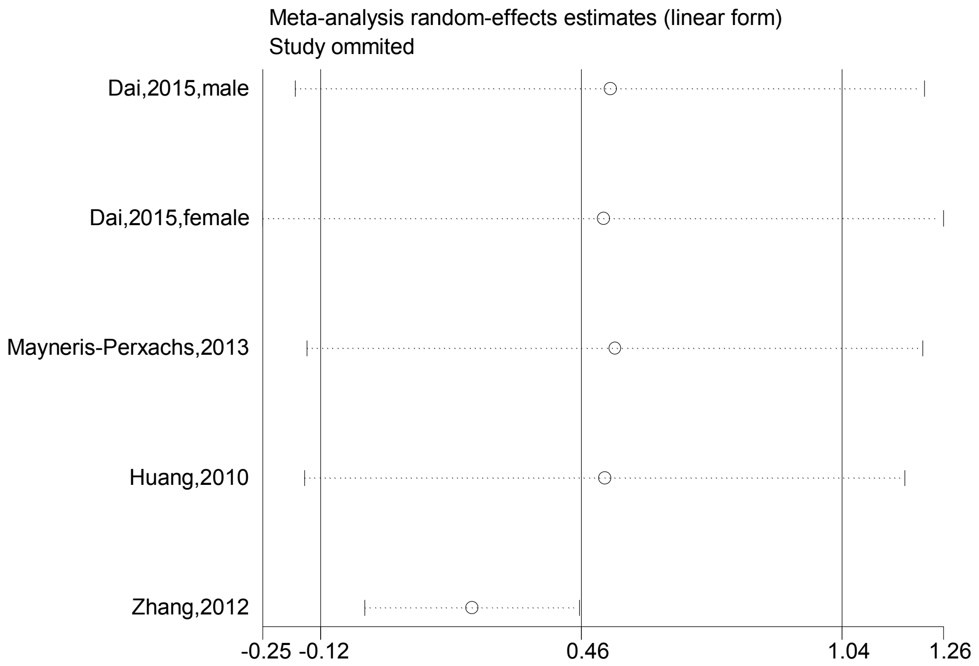

Figure S11 Sensitivity analysis for ALA associated with MetS risk

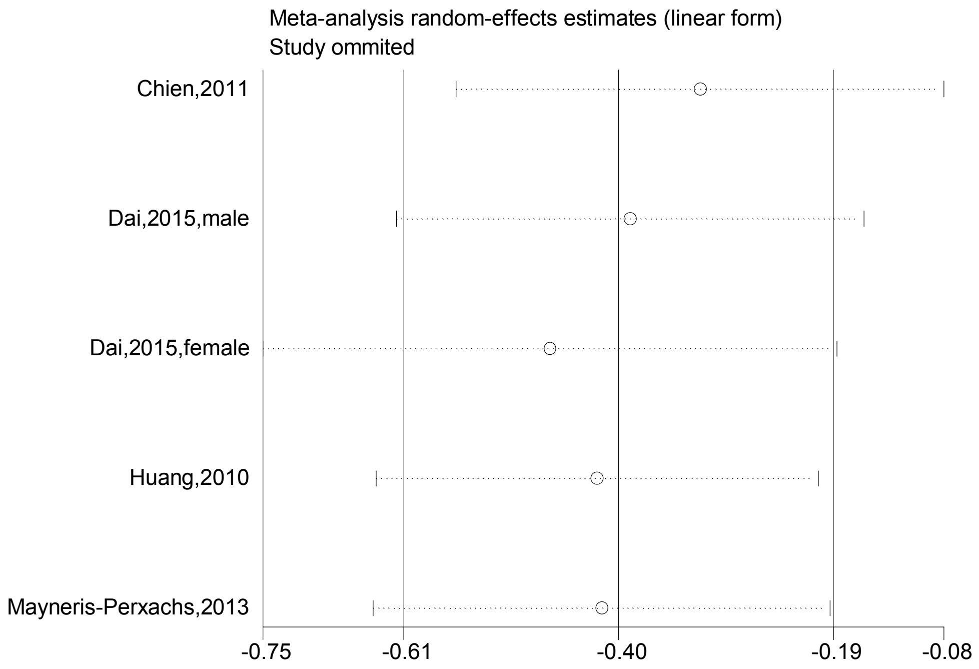

Figure S12 Sensitivity analysis for EPA associated with MetS risk

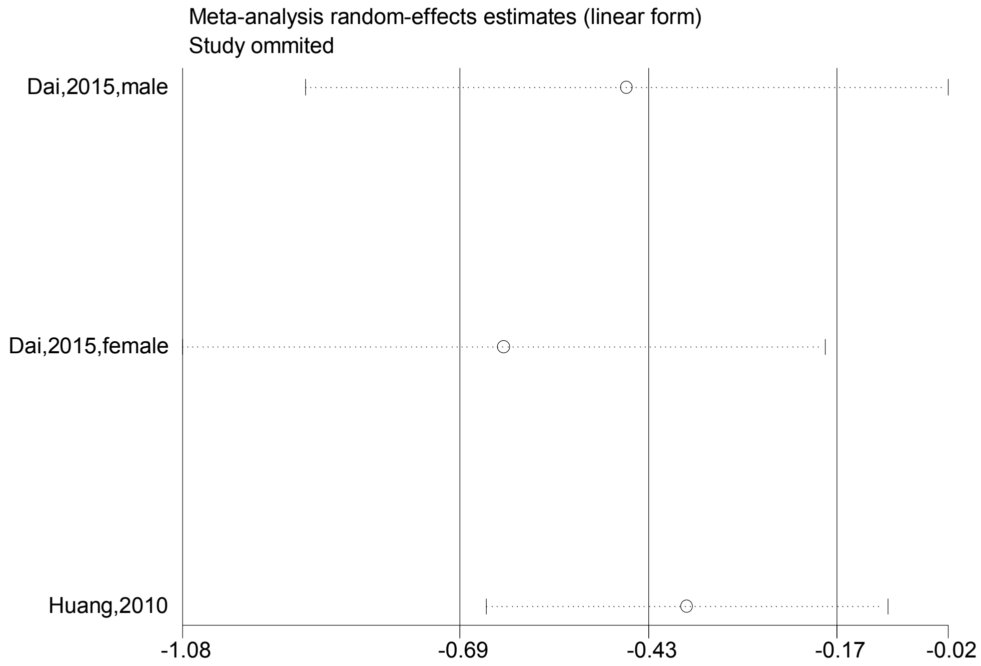

Figure S13 Sensitivity analysis for DPA associated with MetS risk

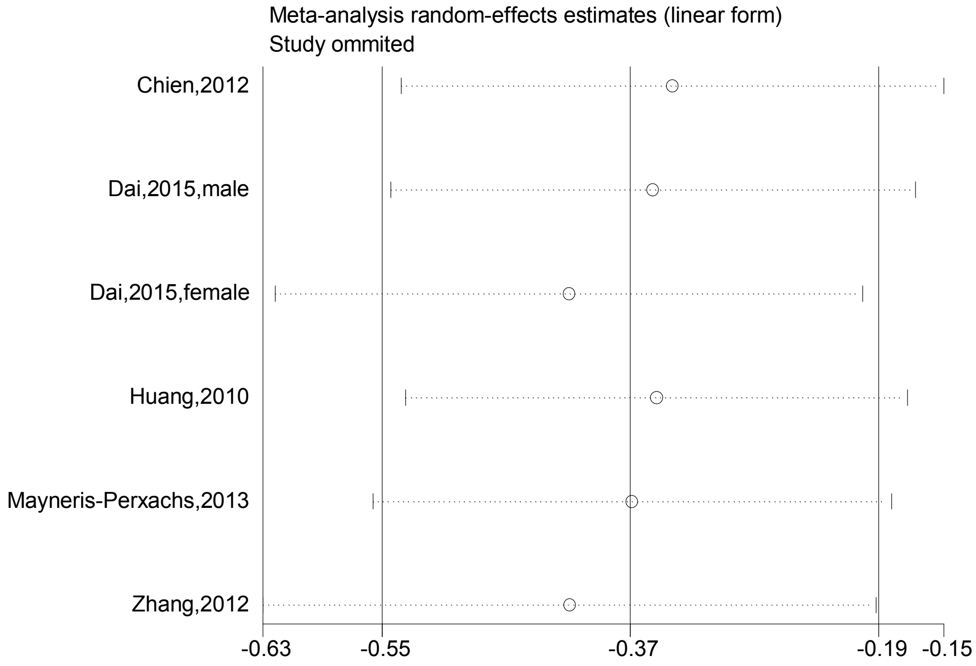

Figure S14 Sensitivity analysis for DHA associated with MetS risk

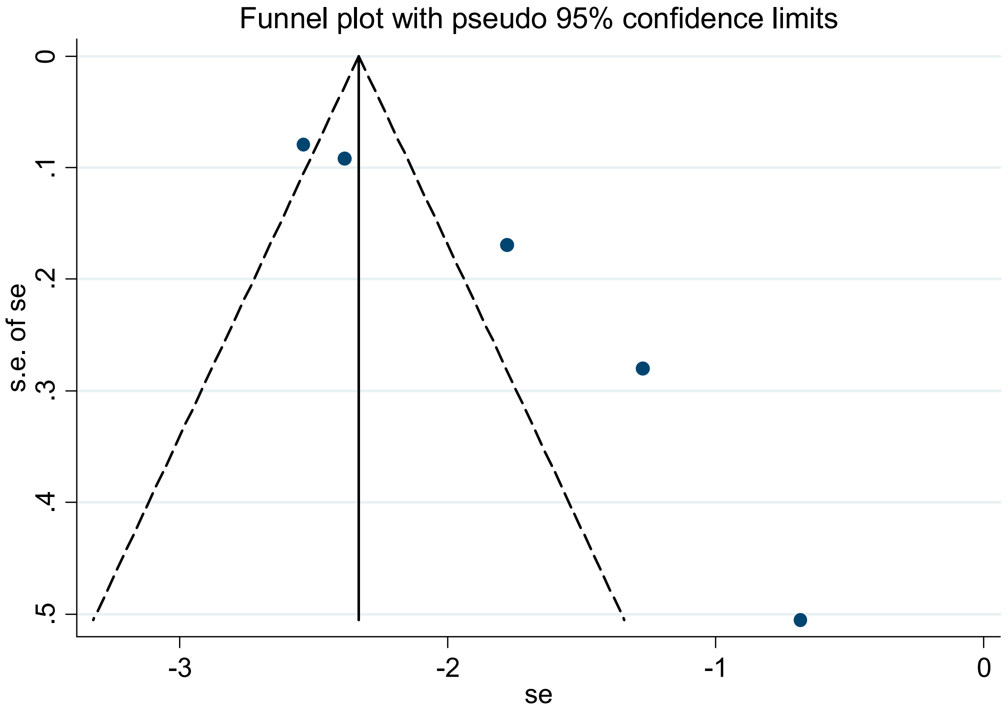

Figure S15 Funnel plot for circulating n-3 PUFAs associated with MetS risk

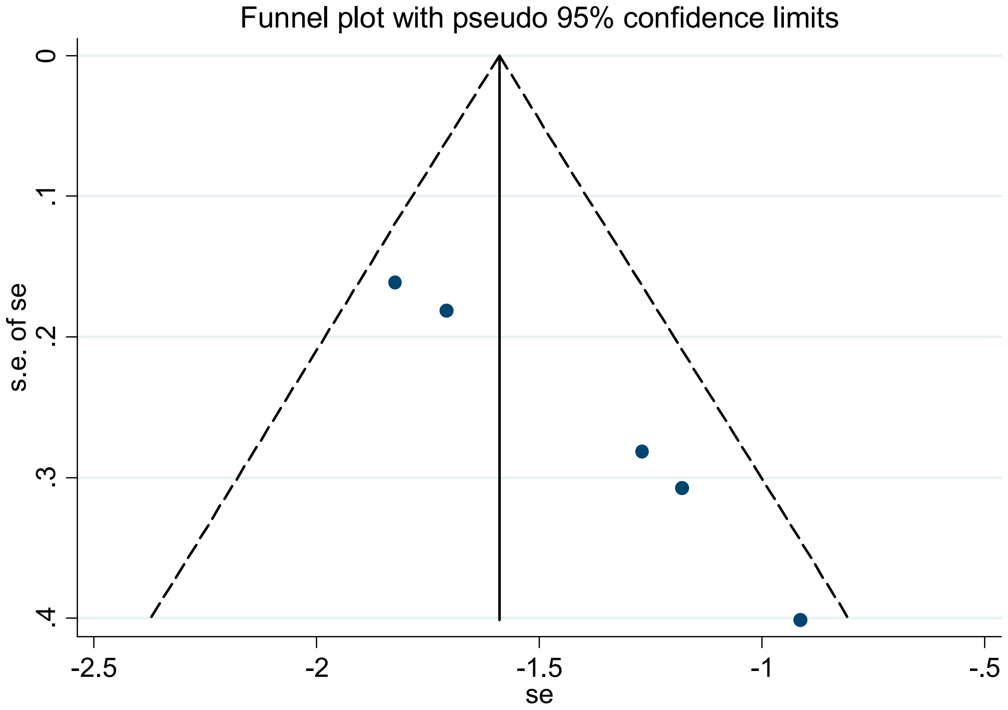

Figure S16 Funnel plot for circulating ALA associated with MetS risk

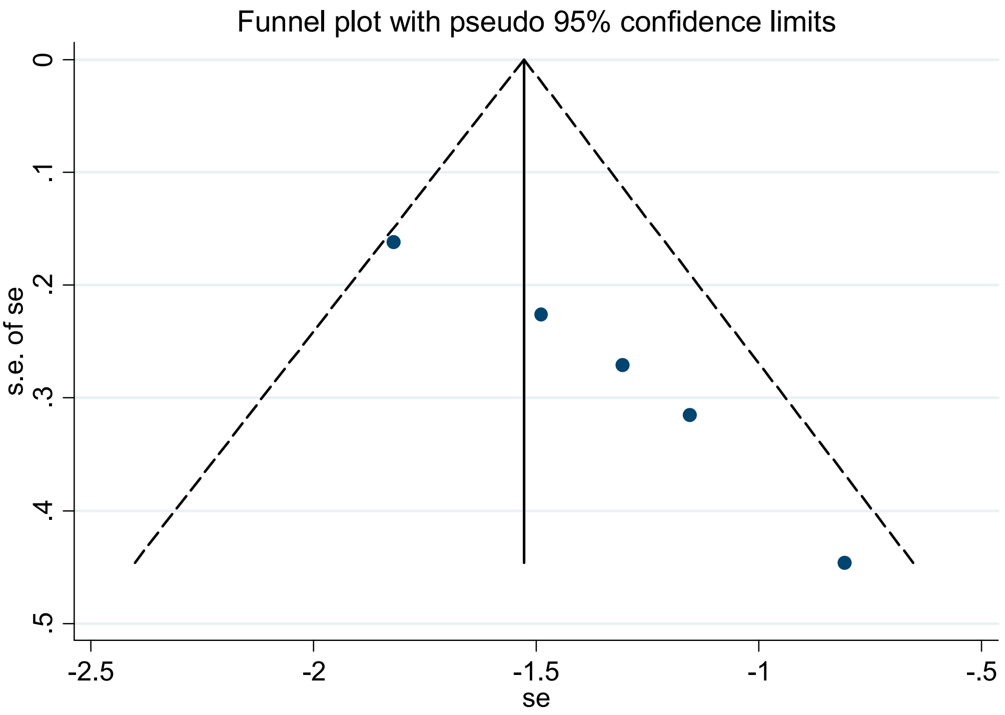

Figure S17 Funnel plot for circulating EPA associated with MetS risk

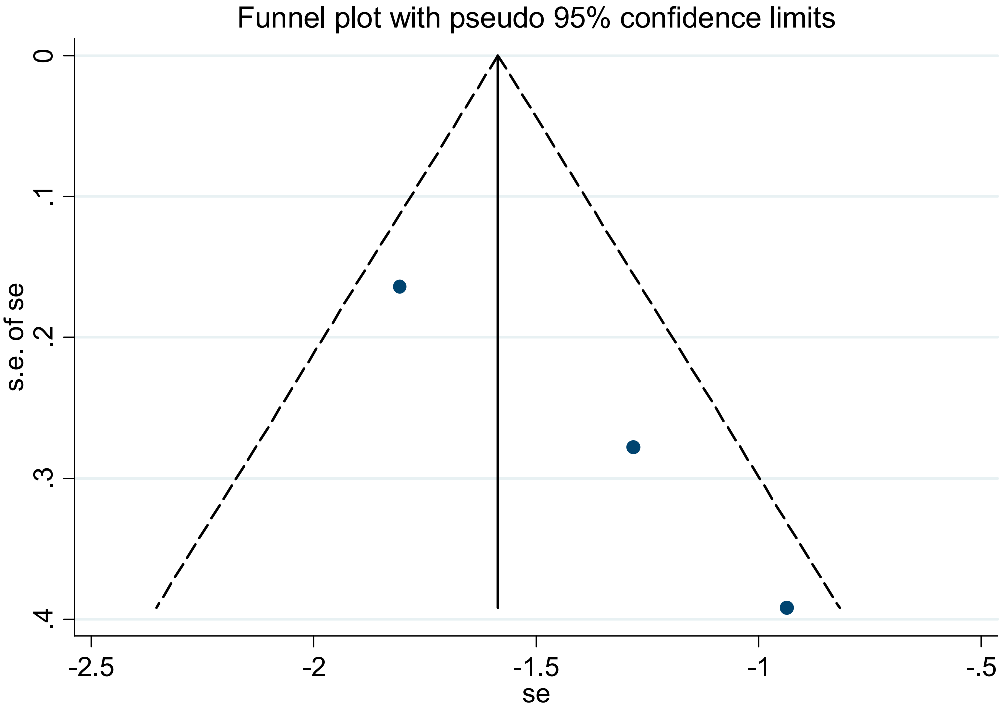

Figure S18 Funnel plot for circulating DPA associated with MetS risk

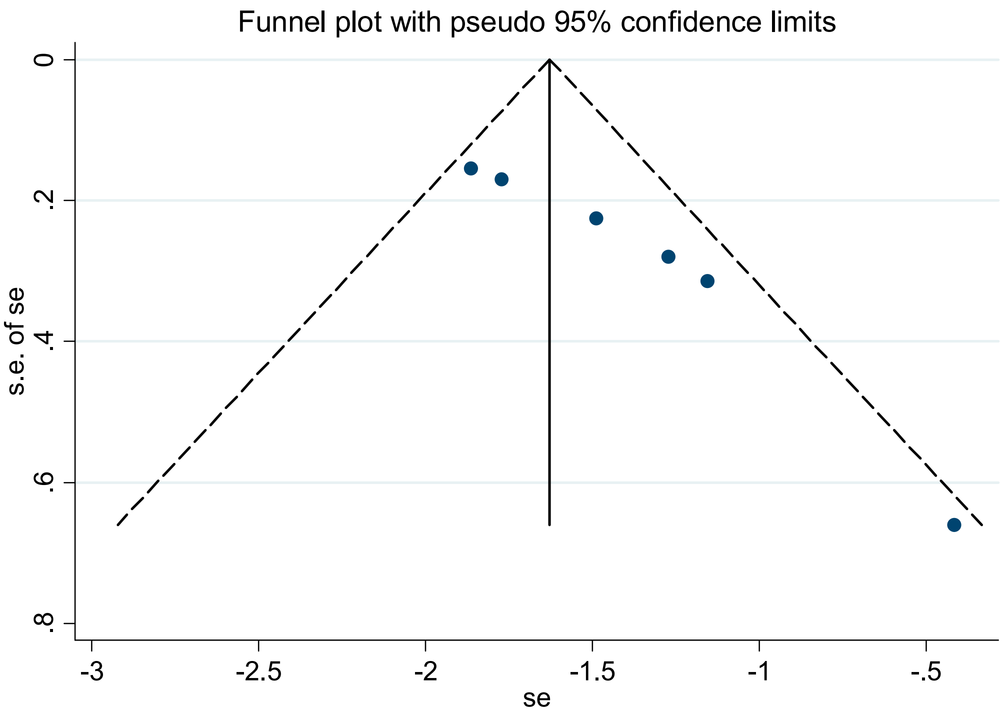

Figure S19 Funnel plot for circulating DHA associated with MetS risk
